# The value of percentile base on computed tomography histogram in differentiating the invasiveness of adenocarcinoma appearing as pure ground-glass nodules

**DOI:** 10.1097/MD.0000000000023114

**Published:** 2020-11-06

**Authors:** Dacheng Hu, Tao Zhen, Mei Ruan, Linyu Wu

**Affiliations:** a Department of Radiology, Affiliated Hangzhou First People's Hospital, Zhejiang University School of Medicine; b Department of Radiology, the First Affiliated Hospital of Zhejiang Chinese Medical University; c The First Clinical Medical College of Zhejiang Chinese Medical University, Zhejiang, Hangzhou, China.

**Keywords:** ground-glass nodule, lung adenocarcinoma, texture analysis, tomography, x-ray computed

## Abstract

To investigate the value of percentile base on computed tomography (CT) histogram analysis for distinguishing invasive adenocarcinoma (IA) from adenocarcinoma in situ (AIS) or micro invasive adenocarcinoma (MIA) appearing as pure ground-glass nodules.

A total of 42 cases of pure ground-glass nodules that were surgically resected and pathologically confirmed as lung adenocarcinoma between January 2015 and May 2019 were included. Cases were divided into IA and AIS/MIA in the study. The percentile on CT histogram was compared between the 2 groups. Univariate and multivariate logistic regression were used to determine which factors demonstrated a significant effect on invasiveness. The receiver operating characteristic (ROC) curve and the area under the curve (AUC) was used to evaluate the predictive ability of individual characteristics and the combined model.

The 4 histogram parameters (25th percentile, 55th percentile, 95th percentile, 97.5th percentile) and the combined model all showed a certain diagnostic value. The combined model demonstrated the best diagnostic performance. The AUC values were as follows: 25th percentile = 0.693, 55th percentile = 0.706, 95th percentile = 0.713, 97.5th percentile = 0.710, and combined model = 0.837 (all *P* < .05).

The percentile of histogram parameters help to improve the ability to radiologically determine the invasiveness of lung adenocarcinoma appearing as pure ground-glass nodules.

## 1 Introduction

Lung cancer is the most common cause of cancer death worldwide,^[1]^ of which lung adenocarcinoma is the most common pathological type. Typically, lung adenocarcinoma manifests as persistent ground-glass nodules, including pure ground-glass nodules (pGGNs) and part-solid nodules. According to the 2015 International Multidisciplinary Classification of Lung Cancer,^[2]^ the pathological subtypes of lung adenocarcinoma can be divided into adenocarcinoma in situ (AIS), minimally invasive adenocarcinoma (MIA), and invasive adenocarcinoma (IA). Different pathological subtypes of lung adenocarcinoma offer different prognosis and clinical management schemes. If adenocarcinoma in situ or micro-invasive adenocarcinoma can be detected early and surgically resected, the 5-year survival rate can approach 100%.^[2,3]^ In particular, wedge or segment resections could provide the same surgical margins with lower chances of postoperative damage. However, the 5-year survival rate of invasive adenocarcinoma (stage I, T1N0M0) is only 74.6%.^[4]^ Generally speaking, pGGN are frequently preinvasive lesions of pulmonary adenocarcinomas. However, it has also been shown that a substantial number of pGGNs eventually prove to be IA. Therefore, it is of great significance to accurately distinguish invasive adenocarcinoma from adenocarcinoma in situ or micro invasive adenocarcinoma appearing as pGGN.

In recent years, quantitative texture analysis has shown good application prospects in the diagnosis of pulmonary nodules.^[5,6]^ However, research about the percentile of histogram parameters to distinguish pGGN invasiveness is still rare. The purpose of this study was to assess the invasiveness of lung adenocarcinoma appearing as pure ground-glass nodules using histogram features based on thin-section CT images and evaluate its diagnostic efficacy.

## 2 Materials and methods

This retrospective study was approved by our institutional review board and the informed consent requirement was waived.

### 2.1 Patients

**Figure 1:**
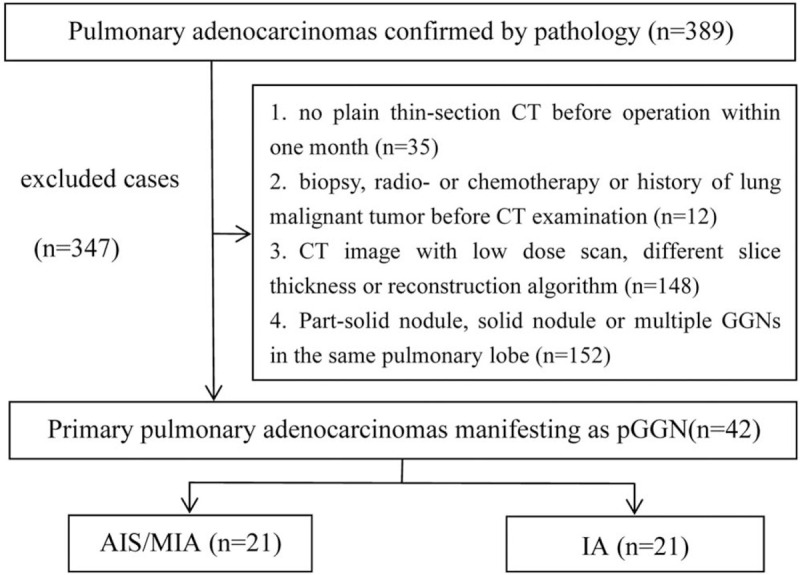
Flowchart of study population.

From January 2015 to May 2019, 389 consecutive pulmonary adenocarcinomas confirmed by operative pathology were reviewed based on the 2015 IASLC/ATS/ERS classification of lung adenocarcinoma in our institution (2). A total of 42 patients with 42 pure ground-glass nodules in our hospital were involved in this study (Fig. 1). All the patients were scanned on the same machine with similar scan parameters. The inclusion criteria for the study were as shown below: the plain thin-section CT was performed in our hospital within 1 month before the operation. The lesion appeared as a pure ground-glass nodule with a maximum diameter within 3 cm on CT imaging; pathological results were obtained by surgical resection. Biopsy, radiotherapy, chemotherapy, or other surgical resection were not performed before CT examination. The exclusion criteria were as follows: no thin-section CT image within 1 month before surgery; lesion appeared as part-solid or solid nodules; different machines or scanning parameters (including voltage, layer thickness, reconstruction algorithm); biopsy, radio-, or chemotherapy performed before CT; multiple pulmonary nodules in the same lung lobe. The patients were divided into group of AIS/MIA and group of IA in our study.

### 2.2 Image acquisition

All images were obtained from the Somatom Sensation 64 (Siemens Healthcare, Germany) CT scanner at our institution. The detailed scanning parameters were as follows: tube voltage 120 kV, tube current automatic modulation, pitch 1.4, collimation 0.6 × 64 mm; reconstruction layer thickness 0.75 mm; layer interval 0.5 mm; matrix, 512 × 512; the reconstruction convolution function B31f.

### 2.3 Visual evaluation and histogram feature extraction

**Figure 2:**
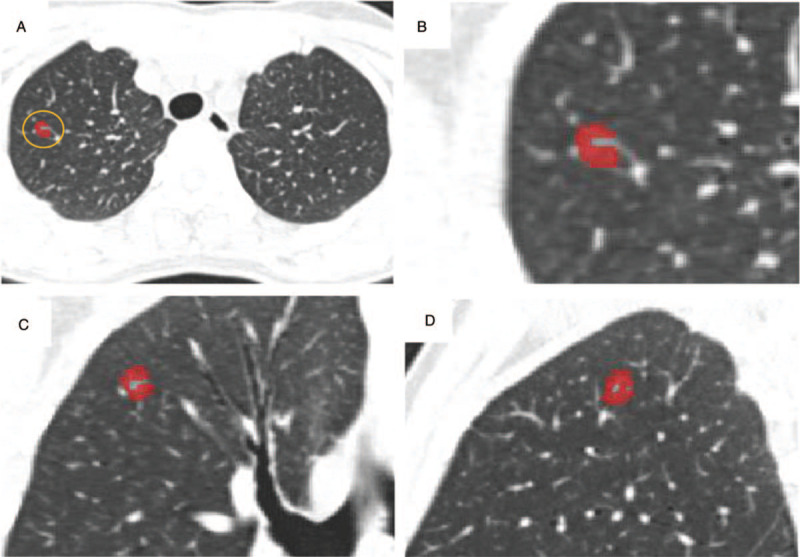
A–D, The region of interest (ROI) of pure ground glass nodules in the right upper lobe of the same patient in the transverse, coronal, and sagittal position, respectively. Postoperative pathology confirmed that it was microinvasive adenocarcinoma.

All images were transmitted to GE PACS for observation. The pGGN was defined as a focal nodular lesion of increased attenuation without internal solid component on a high-resolution CT with a lung window (window width: 1300 HU, window level: –600 Hu). Without knowing the pathological results, 2 radiologists with 3 and 10 years of diagnosis experience segmented the nodule manually by delineating the GGN's margin, layer by layer, on all axial images, excluding large vessels, bronchi, and vacuoles by ITK-SNAP 3.6.0 software (www.itksnap.org) (Fig. 2).

The original thin-slice CT images and the region of interests (ROIs) were imported into A.K. Analysis software (Analysis-Kinetics, GE, Shanghai, China) for histogram feature extraction. Subsequently, the invasiveness of all the pGGN was evaluated by 2 radiologists (with 3 and 10 years of experience in imaging diagnosis) based on CT images without known the pathology. A consensus was reached by discussion in case of discrepant interpretations.

### 2.4 Statistical analysis

Statistical analysis was performed using SPSS 21.0 software (Armonk, New York, USA). The independent *t* test was used for continuous variables, and the chi-square test was used for categorical variables of clinical data. The features were kept as input variables for the multivariate logistic regression analysis with the backward stepwise elimination mode, removing variables based on the likelihood ratio statistics with *P* > .05. The receiver operating characteristic (ROC) curve was plotted for the histogram parameters and the area under the curve (AUC) was calculated. An AUC value of 0.5 to 0.7 indicated a moderate diagnostic efficiency, 0.7 to 0.9 suggested good diagnostic efficiency, and >0.9 suggested excellent diagnostic efficiency. *P* values <.05 considered statistically significant.

## 3 Results

### 3.1 Basic case information

**Figure 3:**
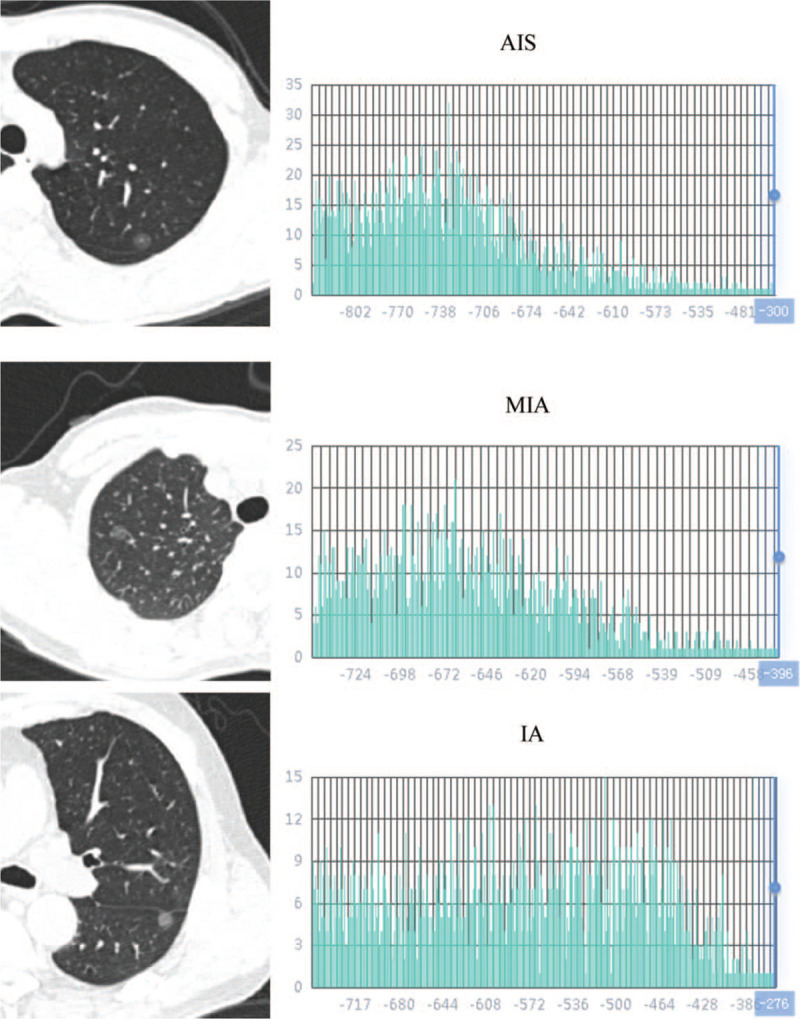
CT image, histogram distribution of CT attenuation value of AIS, MIA, IA. As compared with histograms of AIS and MIA, those of IA show increased values in the 25th, 55th, 95th, and 97.5th percentile (Percentile25: –814.7, –786.9, –748.4; Percentile55: –750.9, –711.3, –610.5; Percentile95: –632.6, –587.9, –435.9; Percentile97.5: –606.5, –567.3, –413.2). AIS = adenocarcinoma in situ, CT = computed tomography; IA = invasive adenocarcinoma, MIA = micro invasive adenocarcinoma.

A total of 42 cases of lung adenocarcinoma were included in this study, including 7 cases of adenocarcinoma in situ, 14 cases of micro invasive adenocarcinoma, and 21 cases of invasive adenocarcinoma (Fig. 1). There was no significant difference in sex, age, and location between the 2 groups of AIS/MIA and IA. The difference was statistically significant in visual evaluation and diameter. The average diameter of invasive adenocarcinoma was significantly larger than that of AIS/MIA (Table 1). CT image and histogram distribution of CT attenuation value were showed in the Fig. 3.

**Table 1:** Basic clinical information of the patients and visual evaluation results of AIS/MIA and IA.

Clinical data	AIS/MIA (n = 21)	IA (n = 21)	*P* value
Sex			.172
Man	8 (38.09%)	4 (19.05%)	
Female	13 (61.90%)	17 (80.95%)	
Age, y	53.71 ± 13.99	56.67 ± 11.49	.696
Location			.486
Right upper lobe	11 (52.38%)	10 (47.61%)	
Right middle lobe	0 (0%)	2 (9.52%)	
Right lower lobe	4 (19.05%)	2 (9.52%)	
Left upper lobe	4 (19.05%)	6 (28.57%)	
Left lower lobe	2 (9.52%)	1 (4.76%)	
Visual evaluation			.002
Absent	1 (4.76%)	10 (47.61%)	
Present	20 (95.24%)	11 (52.38%)	
Diameter	10.10 ± 4.73	12.38 ± 4.66	.027

### 3.2 CT histogram feature extraction and selection

**Figure 4:**
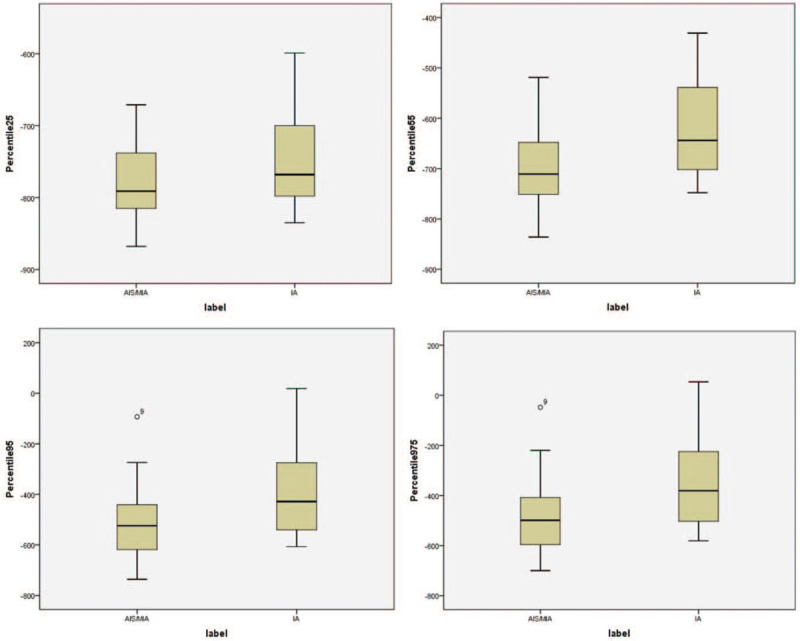
Four set of boxplots visually showed the 4 histogram parameters (25th percentile, 55th percentile, 95th percentile, 97.5th percentile) in AIS/MIA and IA. AIS = adenocarcinoma in situ, IA = invasive adenocarcinoma, MIA = micro invasive adenocarcinoma.

Logistic regression analysis was conducted to find better predictors in distinguishing 2 pathologic subtypes. After the 24 histogram features were extracted, 14 histogram features were significantly associated with invasiveness by univariate logistic regression (*P* < .05, Table 2). Features with *P* < .05 in univariate logistic regression were entered into the multiple logistic regression analysis. Multivariate backward stepwise logistic regression analysis revealed 25th percentile (odds ratio [OR] = 0.635, 95% confidence interval [CI] = 0.422–0.954), 55th percentile (OR = 1.652, 95% CI = 1.074–2.542), 95th percentile (OR = 0.836, 95% CI = 0.725–0.964), 97.5th percentile (OR = 1.14, 95% CI = 1.023–1.270) were independent differentiating factors (all *P* < .05) (Table 2). These 4 parameters (25th percentile, 55th percentile, 95th percentile, 97.5th percentile) were incorporated to develop the combined model and feature with *P* > .05 was eliminated. The box plot of the 4 parameters intuitively showed that the parameters of the invasive adenocarcinoma were higher than the AIS/MIA (Fig. 4).

**Table 2:** Results of univariate and multivariate logistic regression analysis of histogram parameters.

				Multivariate logistic regression	
				
Histogram parameters	AIS/MIA	IA	Univariate logistic regression	OR (95%CI)	*P* value
25th percentile	−709.81 ± 76.43	−645.29 ± 93.92	0.032	0.635 (0.422–0.954)	.029
45th percentile	−724.90 ± 71.01	−666.96 ± 87.76	0.048	NA	NA
50th percentile	−709.81 ± 76.43	−645.29 ± 93.91	0.032	NA	NA
55th percentile	−694.05 ± 83.75	−622.78 ± 101.12	0.023	1.652 (1.074–2.542)	.022
60th percentile	−678.14 ± 90.47	−600.22 ± 109.26	0.023	NA	NA
65th percentile	−660.71 ± 98.80	−576.98 ± 118.66	0.019	NA	NA
70th percentile	−642.33 ± 108.43	−544.19 ± 126.16	0.019	NA	NA
75th percentile	−623.33 ± 118.75	−530.35 ± 134.12	0.016	NA	NA
80th percentile	−602.28 ± 127.97	−506.10 ± 142.68	0.021	NA	NA
85th percentile	−579.09 ± 137.79	−478.09 ± 151.12	0.020	NA	NA
90th percentile	−549.71 ± 149.09	−445.71 ± 157.52	0.023	NA	NA
95th percentile	−507.29 ± 164.31	−397.61 ± 165.44	0.018	0.836 (0.725–0.964)	.014
97.5th percentile	−471.76 ± 172.54	−353.10 ± 167.54	0.020	1.14 (1.023–1.270)	.018

### 3.3 Performance of histogram feature and combined model

**Figure 5:**
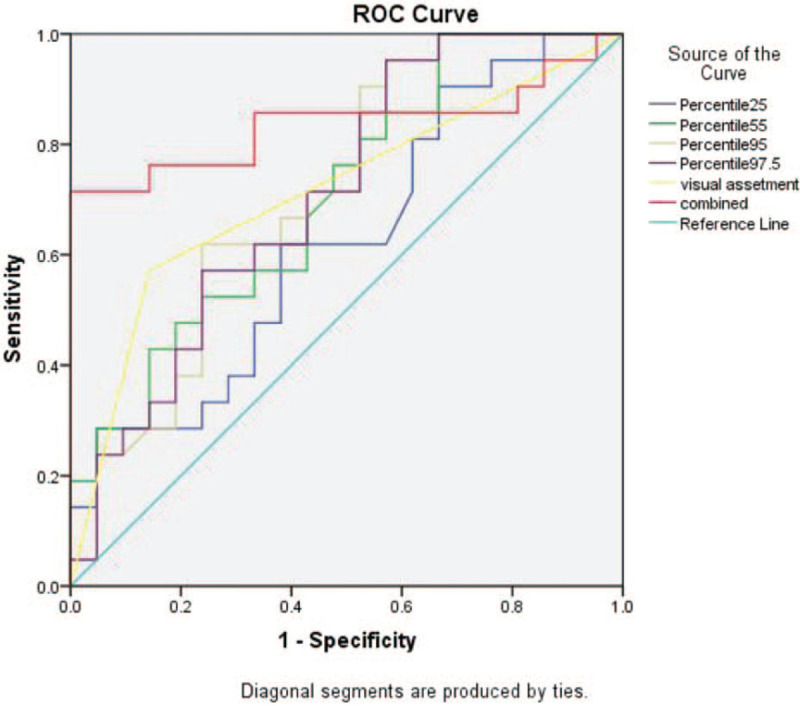
ROC curves of 4 histogram parameters (25th percentile, 55th percentile, 95th percentile, 97.5th percentile), combined model, and visual evaluation in identifying lung adenocarcinoma. ROC curve = the receiver operating characteristic curve.

The 25th percentile, 55th percentile, 95th percentile, 97.5th percentile was jointed as combined model. The ROC curves of the 4 histogram parameters (25th percentile, 55th percentile, 95th percentile, 97.5th percentile), the combined model and visual evaluation of were shown in Fig. 5. All parameters obtained a certain diagnostic value. The AUC values were as follows: 25th percentile, 0.693; 55th percentile, 0.706; 95th percentile, 0.713; 97.5th percentile, 0.710; combined model, 0.837, and visual evaluation, 0.738 (Table 3). The diagnostic sensitivity and specificity of 25th percentile, 55th percentile, 95th percentile, 97.5th percentile, and visual assessment were 76.2%, 52.4%; 71.4%, 52.4%; 61.9%, 76.2%; 71.4%, 57.1%; 61.9%, 85.7%, respectively (Table 3).

**Table 3:** The performance 4 histogram parameters, combined model, and visual evaluation to differential the IA and AIS/MIA.

Parameter	Sensitivity	Specificity	AUC	*P* value
Visual evaluation	0.619	0.857	0.738	.008
25th percentile	0.762	0.524	0.693	.032
55th percentile	0.714	0.524	0.706	.022
95th percentile	0.619	0.762	0.713	.018
97.5th percentile	0.714	0.571	0.710	.020
Combined	0.762	0.857	0.837	≤.001

## 4 Discussion

With the expansion of low dose computer tomography for chest imaging, the detection rate of pulmonary nodules, including ground-glass nodules is increasing. However, it was difficult to assess invasiveness of the overall lesion before surgery. In addition, the traditional CT evaluation is limited by subjective human factors and experience, which makes it difficult to accurately identify the invasiveness of ground-glass nodules, especially pure ground-glass nodules.^[7–10]^

We demonstrated that the percentile parameters of histogram based on thin-section CT images enabled the differentiation of IA from AIS or MIA. The results showed that all 4 histogram parameters have moderate diagnostic value (all AUC > 0.7), and the combined model with 4 histogram parameters showed an even greater diagnostic value (AUC > 0.8). The AUC of the combined model is also higher than the visual assessment (AUC = 0.738). Many studies have reported that quantitative CT texture parameters can help identify pathological subtypes of GGN.^[5,11–16]^ Yang et al^[15]^ studied that histogram parameters such as the 100th percentile or 90th percentile can help identify the invasiveness of ground-glass nodules.^[16]^ Our study showed that the 25th percentile, 55th percentile, 95th percentile, and 97.5th percentile could help to distinguish adenocarcinoma from AIS/MIA from IA presented as pure ground-glass nodules. In addition, the percentile of IA was significantly higher than that of AIS/MIA, which is consistent with previous reports.^[15–17]^ This may indicate that the density of IA is often higher than that of AIS/MIA for pure ground glass nodules. Moreover, combining the 4 histogram parameters, the diagnostic efficiency (AUC = 0.837) is higher than that of the individual histogram parameters. These results showed that the combined histogram parameters can improve classification of lung adenocarcinoma of pure GGNs and it provided higher discriminatory ability than that with visual assessment from conventional CT imaging.

Studies have shown that ground-glass nodules >10 mm have a higher possibility of manifesting as invasive adenocarcinoma.^[17,18]^ Our results showed similar findings for the average diameter of invasive adenocarcinoma. In our research, the average diameter of invasive adenocarcinoma was 12.38 mm. While the average diameter of AIS/MIA was 10.10 mm, which is also >10 mm. This maybe the reason that we include the MIA into this group and most cases in the group of AIS/MIA was MIA (the ratio: 14/21). Wu et al^[19]^ found that vascular changes, poorly defined margins, and a clear tumor–lung interface were significant predictive factors for invasive lesions of pGGNs <10 mm. The AUC of the vessel changes, margin, and tumor–lung interface yielded relatively low diagnostic value (all AUC <0.7). Compared with our study, the diagnostic efficacy of visual evaluation reached a moderate diagnostic efficiency (AUC, 0.738). For pure ground-glass nodules, the morphological characteristics of traditional CT including lobulation, spiculation, and pleural indentation are often not obvious, especially for pGGN <10 mm. Therefore, it was difficult to identify the degree of invasiveness of lung adenocarcinoma by traditional CT features alone. This study also showed that the diagnostic efficiency of the combined model is higher than that of visual evaluation.

There were several limitations in our research. First, as a single-center retrospective study, potential selection bias may exist. For instance, only pGGNs resected by surgery were included, while the pGGNs managed with follow-up were excluded. Second, to prevent the influence of the machine and scanning parameters in this study, the same machine with same scanning parameters were selected, which resulted in fewer cases for this study. Therefore, further sample size is needed for data validation. Finally, the ROI of GGN was manually drawn. As such, some small vessels or bronchi may not be removed completely.

## 5 Conclusions

In conclusion, CT histogram features such as percentile can help differentiate IA from AIS/MIA of pGGN. The combined model can potentially improve the diagnostic ability of invasive adenocarcinomas using non-invasive and readily available methods.

## Acknowledgments

The authors acknowledge the statistician Ge Zhou in our institution.

## Author contributions

**Conceptualization:** Linyu Wu.

**Data curation:** Dacheng Hu, Mei Ruan.

**Methodology:** Linyu Wu.

**Software:** Dacheng Hu, Tao Zhen, Linyu Wu.

**Supervision:** Linyu Wu.

**Validation:** Dacheng Hu, Tao Zhen.

**Writing – original draft:** Dacheng Hu, Mei Ruan.

**Writing – review & editing:** Dacheng Hu, Linyu Wu.
